# Role of the NeuroCuple™ Device for the Postoperative Pain Management of Patients Undergoing Unilateral Primary Total Knee and Hip Arthroplasty: A Pilot Prospective, Randomized, Open-Label Study

**DOI:** 10.3390/jcm12237394

**Published:** 2023-11-29

**Authors:** Jacques E. Chelly, Brian A. Klatt, Yram Groff, Michael O’Malley, Hsing-Hua Sylvia Lin, Senthilkumar Sadhasivam

**Affiliations:** 1 Department of Anesthesiology and Perioperative Medicine, University of Pittsburgh, Pittsburgh, PA 15261, USA; hsl26@pitt.edu (H.-H.S.L.); sadhasivams@upmc.edu (S.S.); 2 Department of Orthopaedic Surgery, University of Pittsburgh, Pittsburgh, PA 15213, USA; klattba@upmc.edu (B.A.K.);

**Keywords:** total knee arthroplasty, total hip arthroplasty, pain, nanotechnology, opioids

## Abstract

Background: The potential effectiveness of the non-pharmacological and nanotechnology-based NeuroCuple™ device in reducing postoperative surgical pain and opioid consumption remains unknown. Methods: This randomized controlled open-label study was conducted in patients undergoing a primary unilateral total knee or total hip arthroplasty. In the recovery room, patients were randomized to receive either standard of care (control group) or standard of care plus two NeuroCuple™ devices. The outcome variables included pain and opioid consumption (oral morphine equivalent, OME in milligrams). Results: A total of 69 patients were randomized to either the NeuroCuple™ group (n = 38) or the control group (n = 31). Use of the NeuroCuple™ devices was associated with a significant 34% reduction in pain at rest (means of area under the curve: 6.3 vs. 9.5; *p* = 0.018) during postoperative days 1–3. Opioid consumption was reduced by 9%. More importantly, use of the NeuroCuple™ devices reduced the number of patients requesting an opioid prescription following discharge from the hospital by 52% (26% vs. 55%, *p* = 0.016). Conclusions: Our data suggest that the NeuroCuple™ device may be an effective non-pharmacological alternative to opioids to manage postoperative pain following unilateral arthroplasty due to its ability to reduce postoperative opioid use.

## 1. Introduction

In 2017, it was estimated that the United States spent over USD one trillion annually on opioid addiction [1,2,3]. Major surgery, including knee and hip arthroplasty, has been demonstrated to increase the risk for opioid use disorder (OUD) because of the associated high perioperative opioid requirement [4,5]. Other risk factors for developing OUD following surgery include preoperative mood disorders, history of substance abuse disorders, preoperative use of opioids, Black race, and Medicaid eligibility [5,6]. The current opioid crisis has led to a renewed interest in non-pharmacologic approaches to perioperative pain management as an alternative to opioids. This is especially important in the era of COVID-19, because evidence shows an increase in the number of opioid use disorder cases and associated deaths by overdose related to COVID-19 [7].

It is also well established that administration of opioids is associated with adverse effects such as post-operative nausea and vomiting, immunosuppression, constipation, and, in some cases, death by respiratory depression and/or hypotension [8,9,10,11], leading to an increase in ICU admissions, delays in recovery, and delays in discharge from the hospital. Recent data indicate that the risk of OUD following surgery is around 5–15% [4].

**Figure 1:**
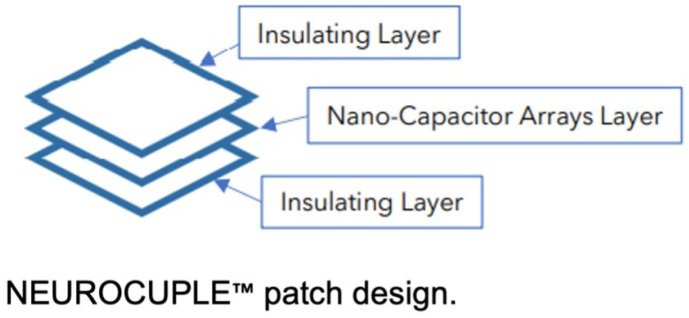
The NeuroCuple^TM^ device (nCap patch) three-layer design, including two outside thin layers of medical grade material that are flexible, durable, and latex-free and an internal layer containing billions of entrapped nanocapacitors.

Recently, the NeuroCuple™ device (nCap Me3dical LLC, Heber City, UT, USA) was developed based on nanotechnology. The NeuroCuple™ device is comprised of three layers, including two outside thin layers of medical grade material that are flexible, durable, and latex-free and an internal layer containing millions of nanocapacitors (Figure 1). An observational study suggested that the NeuroCuple™ device may be effective in reducing pain associated with arthritis [12]. However, the potential effectiveness of the NeuroCuple™ device in reducing postoperative surgical pain and the associated requirement for opioids remains unknown. Therefore, we hypothesized that the NeuroCuple™ device might potentially reduce postoperative pain and opioid consumption in patients undergoing primary and unilateral primary total hip arthroplasty (THA) or total knee arthroplasty (TKA). These types of surgery were chosen because major orthopedic surgery has been identified as a type of surgery associated with the highest risk of postoperative opioid use disorder [4].

## 2. Materials and Methods

### 2.1. Study Desing and Participants

This pilot study was conducted at a single center. It was a prospective, randomized, open-label clinical trial conducted at UPMC (University of Pittsburgh Medical Center) Shadyside Hospital, Pittsburgh, PA, US. Before any eligible patients were recruited and consented, institutional review board approval was obtained (STUDY22010018) and the trial was registered at http://www.ClinicalTrials.gov (NCT 05252858).

Patients undergoing TKA or THA were screened and recruited based on the following criteria. *Inclusion Criteria*: (1) 18 years of age or older. (2) PROMIS^®^ Emotional Anxiety Short Form 8a questionnaire completed with a T-score of ≤57.2. (3) Scheduled for elective primary unilateral TKA or THA. (4) Opioid naïve (use of less than 60 mg OME daily for the last 30 days). (5) Signed written informed consent. *Exclusion Criteria*: (1) Pregnancy. (2) Deemed not suitable for the study at the discretion of the principal investigator. (3) Chronic use of opioids (use of more than 60 mg OME daily for the last 30 days). (4) Active depression, anxiety, or catastrophizing. (5) Active alcoholism or drug abuse. (6) Severe chronic pain condition requiring daily preoperative opioids. (7) Breastfeeding. (8) Concurrent participation in any other clinical trial.

Study recruitment comprised two phases. In phase I, after reviewing and signing an informed consent, each patient was asked to complete a PROMIS^®^ Emotional Anxiety Short Form 8a questionnaire [13] to eliminate the potential interference of anxiety on postoperative pain and opioid consumption, because anxiety has been established as a major determinant of increased postoperative pain and opioid consumption [14,15,16]. In phase II, only patients who were considered not anxious (scoring T-score ≤ 57.2 on the PROMIS^®^ Emotional Anxiety Short Form 8a questionnaire were fully eligible for the study enrollment. Before transferring to the operating room, each patient was also asked to complete the PROMIS^®^ Emotional Depression Short Form 8a [13,14,15,16] and pain catastrophizing questionnaires [17,18,19].

### 2.2. Randomization and Treatment Application

**Figure 2:**
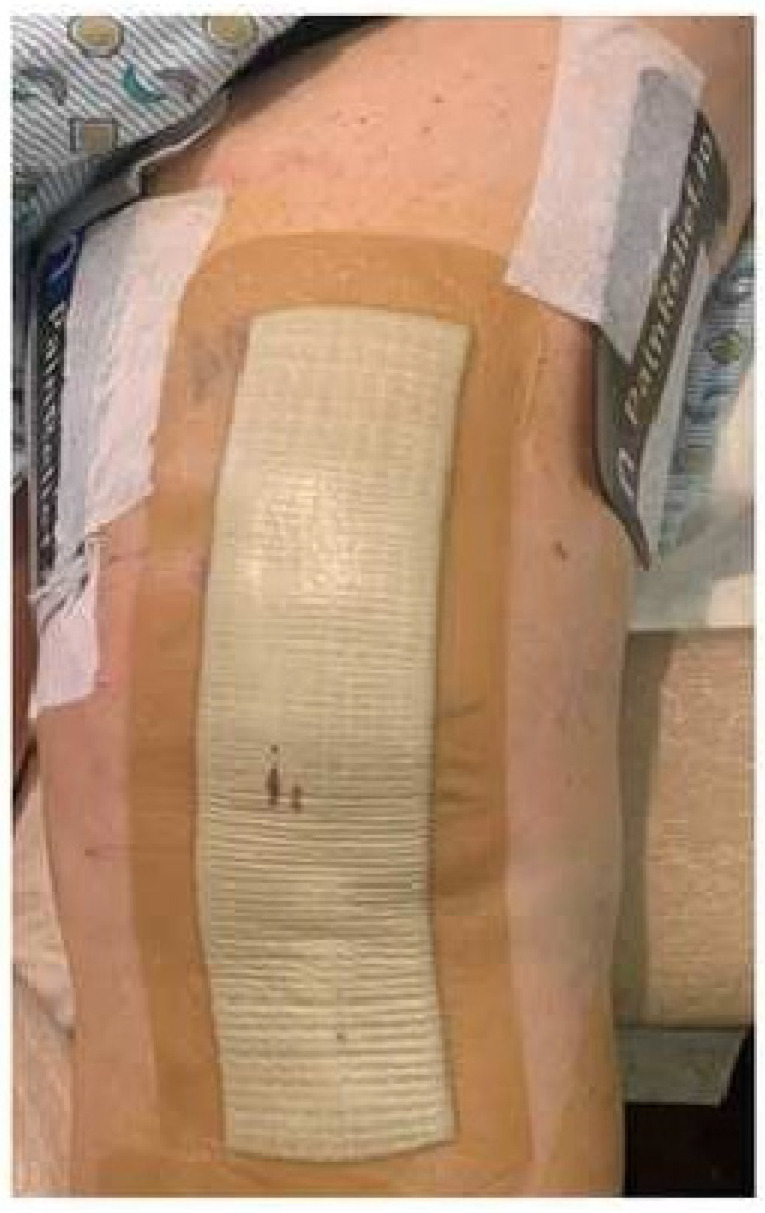
Placement of two NeuroCuple™ devices at the thigh level in the recovery room following total knee arthroplasty.

TKA or THA surgeries were performed under spinal anesthesia and propofol infusion for sedation. In the recovery room, participants were randomized to receive either the standard of care plus the NeuroCuple™ device (treatment group) or the standard of care alone (control group). The randomization of both groups occurred by assigning the participant a subject ID number, and this ID number corresponded to a treatment allocation based on a pre-established electronic simple randomization (1:1) schema. Because of the lack of a placebo NeuroCuple™ device (device without nanocapacitor), the standard of care served as control. Because of the relatively small size of the NeuroCuple™ device, two NeuroCuple™ devices were placed on each side of the thigh in participants randomized to the treatment group. Figure 2 shows the positions of the two NeuroCuple™ devices placed in the recovery room. Each patient in the treatment group was asked to keep the NeuroCuple™ devices for three days. Study follow-up outcome data were assessed on postoperative days (POD) 1, 2, 3, 7, 14, and 30. Clinical outcomes, medications, and patient self-reported outcomes were collected using a web-based data collection program (Research Electronic Data Capture REDCap Cloud Software version 13.8.3 Vanderbilt University, Nashville, TN, USA (RedCap^®^), distributed by the University of Pittsburgh Clinical and Transitional Science Institute, Pittsburgh, PA, USA), in hospital and after discharge.

### 2.3. Primary Outcomes

Pain at rest and during movement were assessed by the Numerical Rating Scale (NRS 0–10, 0 = no pain and 10 = the worst possible pain). Total opioid consumption was expressed in oral morphine equivalents (OME, mg). The number of additional opioid prescriptions requested by the patients after the initial opioid prescription provided at discharge was reported. At the time of discharge from the hospital, each patient was provided with a three-day opioid prescription and instructed to contact the surgeon’s office if they needed another opioid prescription. The number of opioid prescriptions was recorded using the PDMP (Pennsylvania Department of Health Prescription Drug Monitoring Program).

### 2.4. Secondary Outcomes

The time to discharge from the recovery room and the hospital was also collected. T-scores on the PROMIS^®^ Anxiety, Depression, and Sleep Disturbance questionnaires and the pain catastrophizing scores recorded prior to surgery and on postoperative days (PODs) 1, 2, 3, 7, 14, and 30 were also recorded. Functional recovery was assessed by asking the patient the following three questions: Are you able to (a) walk 100 feet (No = 0 or Yes = 1)? (b) Move up five steps (No = 0 or Yes = 1)? (c) Raise your leg (No = 0, some = 1, raise leg completely = 2)? Total scores could vary from 0 to 4. Each patient was asked to complete a survey to rate their overall satisfaction at discharge and on POD 30 on a scale of 0 to 10 (0 = extremely dissatisfied and 10 = the most satisfied). On POD 30, each patient was asked to rate their overall pain treatment satisfaction on a scale of 0 to 10 (0 = totally dissatisfied and 10 = most satisfied).

### 2.5. Statistical Analysis

Power calculation prior to the enrollment indicated that a total of at least 60 patients (30 per group) was required. The total number of patients randomized was 69 to account for two patients who withdrew from the study and to allow randomization of a total of 60 patients who underwent TKA, because most enrolled patients underwent TKA rather than THA (60 TKA vs. 9 THA). Data were analyzed using intention-to-treat analysis. Data are summarized as mean ± standard deviation (SD) for continuous variables and numbers with percentages (%) for categorical variables. The area under the curve (AUC) for pain scores was calculated using the trapezoid rule from PODs 1 to 30, PODs 1 to 3, and PODs 7 to 30. The percent reduction was determined by calculating the difference in means or proportions (treatment group—control group), which was then divided by the mean or proportion in the control group for each outcome. The non-parametric Kruskal–Wallis test was used to determine the differences in the distribution of continuous outcomes, and Pearson’s chi-squared test was used for differences in the proportions for categorical outcomes between the NeuroCuple™ and control groups. The statistical software package SAS (version 9.4; SAS Institute Inc.) was used for the analyses. All tests were two-sided and the alpha level was set at 0.05.

## 3. Results

**Figure 3:**
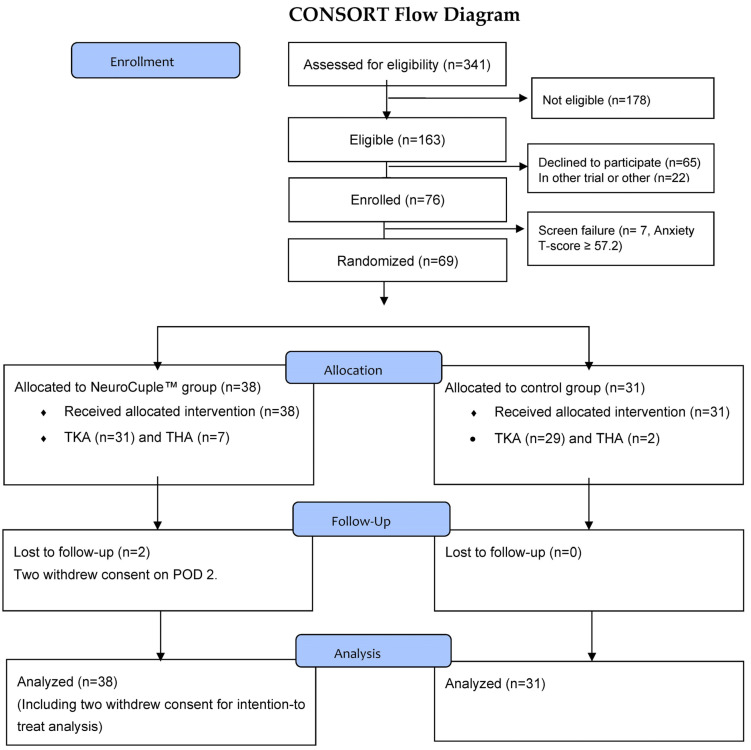
The Consort Flow Diagram.

Of the total 341 patients who were screened, 163 were eligible for the study and 76 signed the informed consent form. The first patient was enrolled on 21 March 2022 and the last patient was enrolled on 8 September 2022. Seven patients were considered a screen failure because their PROMIS^®^ Emotional Anxiety Short Form-8a T scores were ≥57.2 (63.1 ± 4.73). Consequently, 69 patients were randomized (PROMIS^®^ Emotional Anxiety Short Form-8a T scores 46.1 ± 6.97). Among the patients randomized, nine underwent THA and 60 underwent TKA, including two patients who withdrew consent on POD 2 from the treatment group (one THA and one TKA) (Consort Flow diagram; Figure 3).

Table 1 shows the patients’ demographic data (sex, race, weight, height, body mass index), types of surgery, PROMIS^®^ Emotional Anxiety Short Form-8a T-scores, PROMIS^®^ Emotional Depression Short Form-8a scores, PROMIS^®^ Pain Interference scores, pain catastrophizing scores, and initial functional status. Except for the initial PROS^®^ Emotional Anxiety Short Form-8a T scores, which were significantly higher in the NeuroCuple™ group compared with the control group, there were no differences between the groups. Differences in the PROMIS^®^ Emotional Anxiety Short Form-8a T scores were not clinically significant, since both numbers were below the threshold for mild anxiety.

**Figure 4:**
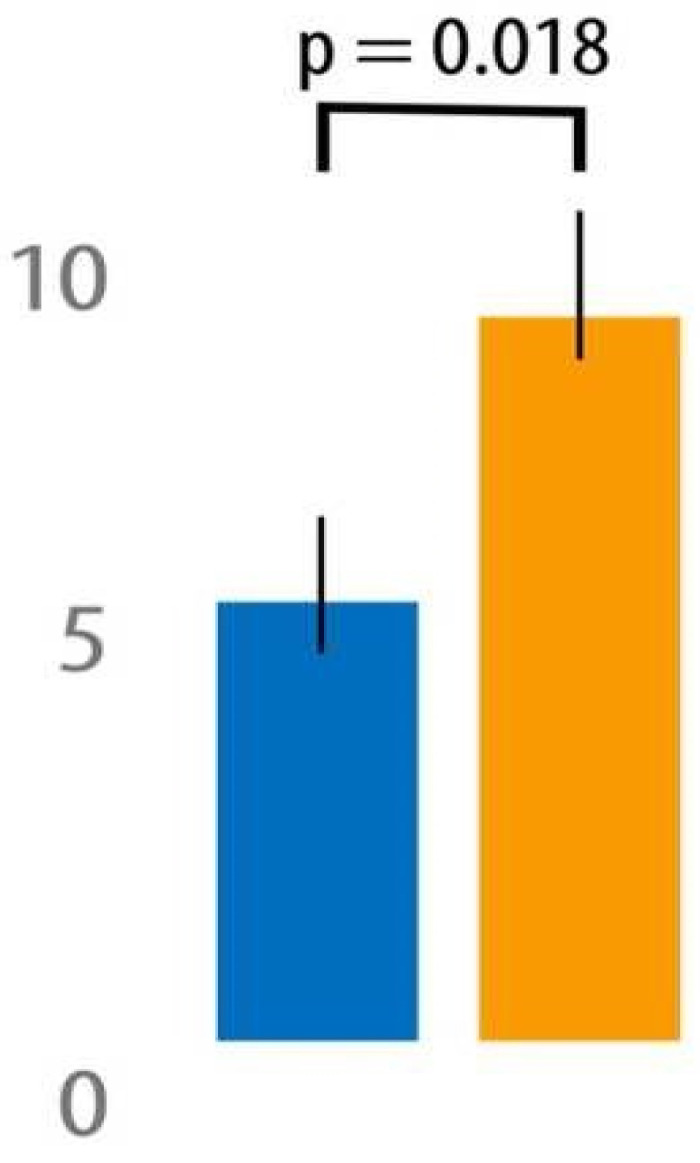
Pain level (AUC) in the NeuroCuple™ group and the control group over the first three postoperative days using the area under the curve (blue = NeuroCuple™ group, orange = control group). *p* < 0.05 was considered significant.

As indicated in Figure 4, between PODs 1 and 3, the use of NeuroCuple™ devices resulted in a significant 34% reduction in pain (AUC between PODs 1 and 3) at rest (6.3 ± 3.7 in the NeuroCuple™ group vs. 9.5 ± 5.6 in the control group; *p* = 0.018) and a 12% reduction in pain during movement (12.0 ± 4.7 in the NeuroCuple™ group vs. 13.7 ± 4.7 in the control group; *p* = 0.12). Opioid consumption was reduced by 6.6 mg in the NeuroCuple™ group (63.6 ± 62.1 OME in mg vs. 70.2 ± 53.8 OME mg; *p* = 0.37). The percentage of patients who did not use opioids during the first three postoperative days was greater in the NeuroCuple™ group (14% in the NeuroCuple™ group vs. 6% in the control group; *p* > 0.05). Similar data were observed for pain and opioid consumption during PODs 7-30 and PODs 1-30 (Table 2).

**Figure 5:**
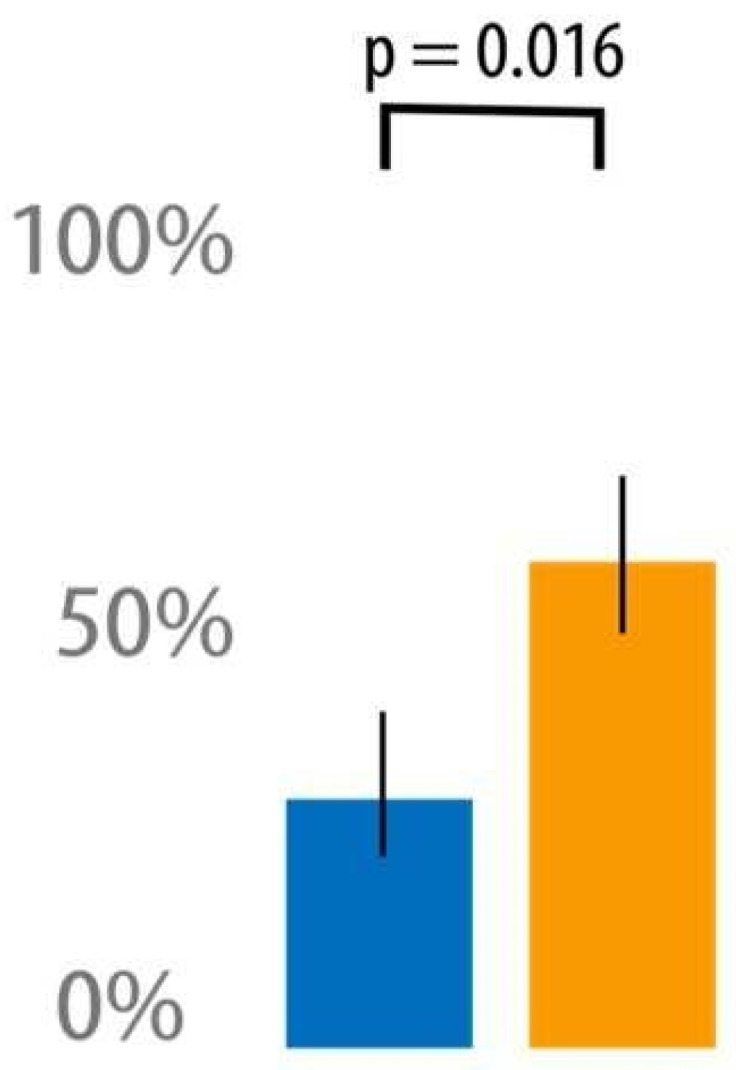
Percentage of patients in the NeuroCuple™ group and control group who called the surgeon’s office and asked for an opioid prescription refill within the first 30 days following surgery. *p* < 0.05 was considered significant. (blue = NeuroCuple™ group, orange = control group).

More importantly, as presented in Figure 5, the use of NeuroCuple™ devices was associated with a 52% reduction in the number of patients requesting opioid prescription refills in the NeuroCuple™ group, as recorded during the first 30 PODs (26% in the NeuroCuple™ group vs. 55% in the control group, *p* = 0.016). the percentage of patients in each group who called the surgeon’s office and asked for an opioid prescription refill within the first 30 days following surgery.

The length of hospital stay in the NeuroCuple™ group was reduced by 23% compared with the control group (*p* = 0.62). Overall patient satisfaction at discharge and on POD 30, as well as satisfaction regarding pain surveyed on POD 30, were similar between the NeuroCuple™ and control groups (Table 2). No local side effects related to the application of the NeuroCuple™ devices for three days were reported by the patients in the treatment group.

## 4. Discussion

Patients undergoing TKA and THA often request opioid refills [20]. Our data suggest that the use of NeuroCuple™ devices represents a potentially very interesting approach to significantly decrease the need for additional opioid prescriptions after discharge from the hospital following TKA and THA. This is especially important in the context of the current opioid crisis and the role that persistent opioid use plays in the development of OUD following surgery in opioid-naïve patients [6]. The frequency of requests for an opioid refill in this population is in the order of 30% and over 80% in patients following TKA [21].

The medical application of nanotechnology is recent and considered to have limitless indications because this technology allows the transport and delivery of nanoparticles, micelles, quantum dots, liposomes, nanofibers, and nanoscaffolds [22,23,24,25,26,27,28,29,30,31,32]. Most medical indications for nanotechnology are nanoscale based to improve drug delivery systems for cancer and chronic wound infection, measure neuronal and cardiac cellular electrical activity and cell-to-cell interactions, and monitor metabolic function at the cellular level. Other techniques, including complementary and alternative techniques such as acupuncture [33,34], hypnosis [35], transcutaneous electrical nerve stimulation [36,37,38], auriculotherapy, and auricular nerve stimulation [39] have been proposed to manage perioperative pain in orthopedics.

In this study, we focused on patients without significant anxiety prior to surgery who were identified by screening with the PROMIS^®^ Anxiety questionnaire. This explained why we only included patients with T-scores ≤ 57.2. We decided to eliminate any central interferences [13,16], since it has been reported that up to 50% of patients undergoing joint arthroplasty have significant anxiety, depression, sleep disorders, catastrophizing, and mood disorders and that these preoperative conditions were associated with an up to 50% increase in postoperative pain and opioid consumption.

The NeuroCuple™ device is an innovative application of nano and quantum technology because it focuses on a macro device containing a very large number of nanocapacitors. The proposed mechanism of action for the NeuroCuple™ device in pain is based on the concept that local trauma is associated with the destruction of biological capacitors. In physiologic conditions, cell membranes act as biological capacitors, leading to the distribution of electrons and electrical charges on each side of cell membranes and the role of the piezoelectric properties they display [40,41,42]. This is especially true for collagen [43,44], because it comprises long protein chains that serve as an insulated material surrounded by nanolayers of ordered water and is present all over the body. In the case of local tissue injury, cell membranes are destroyed. This leads to the release of electrons and electrically charged molecules into the interstitial space. The result is an increase in the local concentration of electrons, charged biological materials, and fluid in the intertidal space. The local pH drops and fluid accumulates, leading to inflammation and pain. The NeuroCuple™ device acts as a mechanical capacitor to re-establish the local electromagnetic equilibrium. The excess fluid is redistributed and local inflammation and pain are reduced. Using a capacitor modem 8040 from MasTec Advanced Technology (Coral Gables, FL, USA), it was determined that the NeuroCuple™ device reading at baseline was 35 pF vs. 455–474 pF when the device was applied at the level of the proposed area following a knee replacement. The increase in capacitance reading validates the proposed mechanism of the NeuroCuple™ device (nCap Medical personal communication).

**Figure 6:**
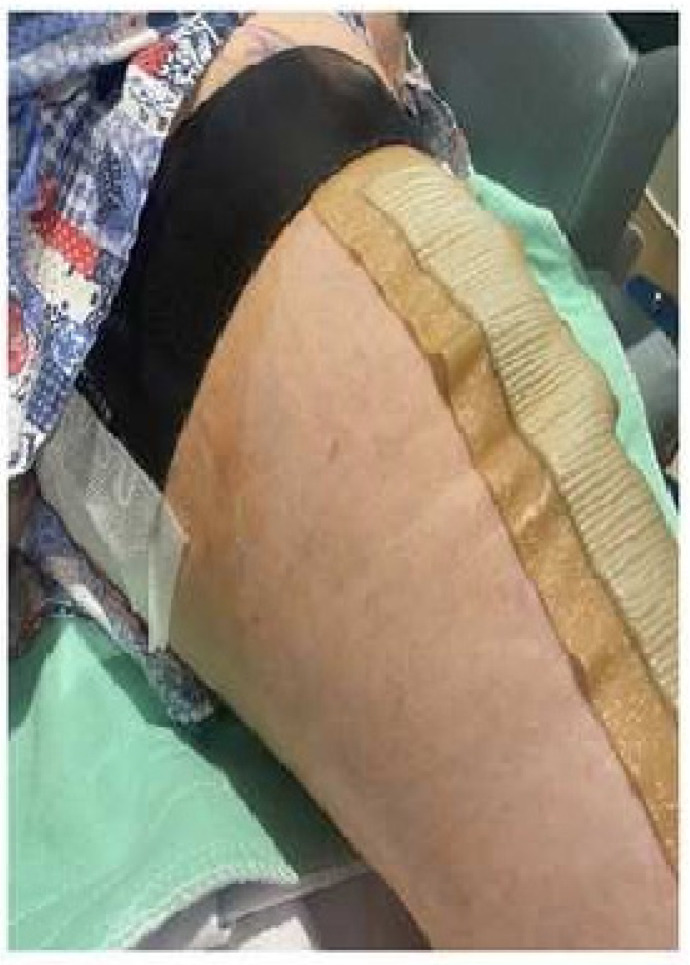
Placement of the newly designed NeuroCuple™ device allowing coverage of the antero-lateral aspect of the thigh following a total knee arthroplasty.

This study should be considered as a pilot study and suggests that nanotechnology may represent an alternative to opioids for the perioperative management of pain following joint arthroplasty. However, the following limitations need to be acknowledged. One, the study did not include a placebo NeuroCuple™ device arm. This was because a placebo NeuroCuple™ device was not available. Second, because the design of the NeuroCuple™ device used at the time of the study was too small to cover the femoral nerve territory, we were required to use two NeuroCuple™ devices for each patient. At our request, nCap Medical has developed a new device. This new NeuroCuple™ device is presented in Figure 6. Third, most patients enrolled in the study underwent TKA. Therefore, the data reported mostly applies to the use of NeuroCuple™ devices in TKA. The statistical analysis performed using only TKA data were similar to those reported using data on both THA and TKA.

## 5. Conclusions

Our study suggests that the NeuroCuple™ device may be a promising non-pharmacologic alternative to manage postoperative pain and reduce the need for opioid prescription refills following joint arthroplasty. This is especially interesting in the context of the current opioid crisis, because the persistent use of opioids following surgery puts patients at risk for OUD. However, additional studies based on a placebo-controlled design and using a longer NeuroCuple™ device are required to confirm the potential value of the NeuroCuple™ device as an alternative to opioid use in patients undergoing joint arthroplasty.

## Figures and Tables

**Table 1:** Baseline characteristics by treatment arm.

	All n = 69	Control n = 31	Treatment n = 38	*p*-Value
Sex				0.47
Male	39 (57%)	19 (61%)	20 (53%)	
Female	30 (43%)	12 (39%)	18 (47%)	
Age (years)	68.7 (8.9)	69.6 (9.0)	68.0 (8.9)	0.45
Race				0.23
White	60 (87%)	25 (81%)	35 (92%)	
African American	7 (10%)	5 (16%)	2 (5%)	
American Indian/Alaska Native	1 (1%)	1 (3%)	0 (0%)	
Asian	1 (1%)	0 (0%)	1 (3%)	
Hispanic	1 (1%)	0 (0%)	1 (3%)	0.36
Weight (kg)	89.6 (14.2)	89.1 (17.8)	89.9 (10.6)	0.82
Height (cm)	169.5 (11.3)	169.0 (11.5)	170.0 (11.4)	0.71
BMI (kg/m^2^)	31.2 (4.7)	30.9 (4.7)	31.4 (4.8)	0.60
Surgery Type				0.14
Total Knee Arthroplasty	60 (87%)	29 (94%)	31 (82%)	
Total Hip Arthroplasty	9 (13%)	2 (6%)	7 (18%)	
PROMIS^®^—Anxiety	46.2 (7.0)	44.1 (7.5)	48.0 (6.0)	0.041
PROMIS^®^—Emotional Distress—Depression	41.3 (6.2)	40.4 (4.7)	41.9 (7.1)	0.57
PROMIS^®^—Sleep Disturbance	49.6 (8.3)	47.4 (9.3)	51.4 (7.0)	0.06
Pain Catastrophizing	6.3 (7.2)	6.8 (8.4)	5.8 (6.1)	0.92
Function	3.6 (0.8)	3.5 (1.0)	3.7 (0.6)	0.68

Mean (SD) for continuous variables and n (%) for categorical variables. BMI: body mass index.

**Table 2:** Outcomes in the NeuroCuple™ treatment group compared with control.

Variable	Control (n = 31)	Treatment (n = 38)	Reduction % Compared with Control	*p*-Value
**Pain at rest AUC**				
	n = 31	n = 36		
POD 1-30	101.5 (75.8)	59.4 (41.6)	41%	0.027
	n = 31	n = 36		
POD 1-3	9.5 (5.6)	6.3 (3.7)	34%	0.018
	n = 31	n = 34		
POD 7-30	75.4 (61.2)	44.0 (28.8)	42%	0.07
**Pain with movement AUC**				
	n = 31	n = 36		
POD 1-30	140.2 (73.7)	114.7 (54.5)	18%	0.12
	n = 31	n = 36		
POD 1-3	13.7 (4.7)	12.0 (4.7)	12%	0.12
	n = 31	n = 34		
POD 7-30	101.5 (61.7)	85.4 (38.2)	16%	0.35
**Total opioid OME (mg)**				
	n = 31	n = 38		
POD 1-30	102.6 (74.0)	82.1 (80.3)	20%	0.15
	n = 31	n = 38		
POD 1-3	70.2 (53.8)	63.6 (62.1)	9%	0.37
	n = 31	n = 36		
POD 7-30	32.4 (41.7)	19.5 (26.5)	40%	0.26
**Opioid refill after POD 3**				
	n = 31	n = 38		
POD 3-30	17 (55%)	10 (26%)	52%	0.016
	n = 31	n = 38		
**Acetaminophen (mg)**				
	n = 31	n = 38		
POD 1-30	10632.7 (4228.2)	9801.3 (5130.1)	8%	0.53
	n = 31	n = 38		
POD 1-3	5944.0 (2622.7)	5750.7 (2805.3)	3%	0.68
	n = 31	n = 35		
POD 7-30	4688.7 (2964.6)	4397.9 (2964.7)	6%	0.62
**Ibuprofen (mg)**				
	n = 31	n = 38		
POD 1-30	616.1 (1814.6)	426.3 (964.1)	31%	0.82
	n = 31	n = 38		
POD 1-3	409.7 (956.2)	189.5 (351.7)	54%	0.98
	n = 31	n = 35		
POD 7-30	206.5 (1011.2)	257.1 (901.7)	(25%)	0.50
**Gabapentin (mg)**				
	n = 31	n = 38		
POD 1-30	391.9 (629.8)	263.2 (341.2)	33%	0.61
	n = 31	n = 38		
POD 1-3	314.5 (375.8)	218.4 (244.8)	31%	0.53
	n = 31	n = 35		
POD 7-30	77.4 (328.3)	48.6 (150.2)	37%	0.84
**PACU min to discharge**	n = 31	n = 38		
	194.4 (72.9)	166.5 (76.7)	14%	0.13
**Hospital hours to discharge**	n = 31	n = 37		
	40.9 (36.2)	31.6 (21.7)	23%	0.62
**Satisfaction with care at discharge**	n = 30	n = 38		
	9.4 (1.6)	9.7 (0.7)	(3%)	0.97
**Satisfaction for care at POD 30**	n = 30	n = 34		
	9.3 (1.7)	9.4 (1.1)	(1%)	0.79
**Satisfaction for pain management at POD 30**	n = 30	n = 33		
	8.1 (2.7)	9.0 (1.8)	(11%)	0.19

AUC: area under the curve; POD: postoperative day; OME: oral morphine equivalent; PACU: post-anesthesia care unit. Mean (SD) for continuous variables and n (%) for categorical variables. The Kruskal–Wallis test or Pearson’s chi-squared test were used to test whether distributions of continuous or categorical outcomes differed by treatment group.

## Data Availability

Data are available on http://www.ClinicalTrials.gov (NCT 05252858).
